# Mental Health Literacy of Chinese College Students: Implications on Mental Health Education in China

**DOI:** 10.7759/cureus.79614

**Published:** 2025-02-25

**Authors:** Wang Chunyan, Ma. Agatha Anne D Guintu

**Affiliations:** 1 Educational Management, Angeles University Foundation, Shandong Huayu Institute of Technology, Angeles, PHL; 2 Educational Management, Angeles University Foundation, Angeles, PHL

**Keywords:** china, college students, mental health education, mental health literacy, mhl

## Abstract

In the face of China's rapid economic development and the increasingly competitive environment, mental health has been a growing public concern. Mental health literacy (MHL), defined as a series of knowledge, beliefs, and abilities that help one understand, cope with, and prevent mental health problems, has been identified as an important step toward mental health promotion especially for vulnerable groups like the youth. College students are at a crucial point in their development and face different challenges that may affect their mental health. This literature review presents the current research status of MHL internationally and in China. It also provides information on important research findings in MHL of foreign and Chinese college students. Implications of MHL on mental health education in China are also explored.

## Introduction and background

In recent years, rapid developments in science and technology have significantly improved productivity and the overall quality of life in China. However, a major social conflict has emerged -- the growing demand for a better life contrasts with uneven and insufficient development, leading to new issues and challenges. First, the family environment has changed, especially impacting the youth. Changes in China's birth rate, as evidenced by the 2023 data, have shown a birth rate of 6.39% and a death rate of 7.87%, resulting in a natural population growth rate of -1.48%. There were also shifts in family structure, characterized by more young people pursuing freedom and living wealthy lives [1]. Most college students of today were born after 2000, grew up in relatively wealthy family environments, and enjoyed more educational resources. However, this made them more likely to feel frustrated and dependent when facing difficulties and challenges. College students who have experienced this have shown new characteristics -- both enjoying the blessings of abundant material life and scientific and technological progress, as well as the various pressures and challenges brought about by social transformation. As a result, mental health problems among university students have become increasingly prominent [2]. Students are in a critical period of growth and are actively learning. They may face unprecedented pressure, have low levels of mental health literacy (MHL), and mental health issues need to be taken seriously [3].

With all these mentioned, it is very important to provide mechanisms to adequately offer college students mental health support. Relevant documents from China's National Health Commission pointed out that one of the most fundamental, economical, and effective measures to improve the mental health of the whole people is by improving their MHL. MHL refers to an individual's personal mental health knowledge, mental health attitudes, and mental health behaviors which include one's coping mechanisms and strategies [4].

This is particularly important for college students as studies have shown that the level of MHL of Chinese college students was found to be relatively low [5]. One key way to improve college students' MHL is mainly through educational intervention [6], specifically, one that relies on a systematic mental health education curriculum system [7]. The positive effect of education on college students' MHL and individual mental health level has been confirmed by domestic and foreign scholars.

This literature review provides an analysis of the present status of MHL research, both foreign and local. It also specifically tackles MHL research among college students in other countries and in China. Understanding their present concerns and needs as well as research gaps has important implications in delivering systematic and evidence-based mental health education in China.

## Review

The concept of MHL

The conceptualization of MHL has gradually developed over the years, from a simplistic and limited view to a more comprehensive construct. Jorm, an Australian scholar, first proposed the concept of MHL, which originally referred to "the knowledge and concepts that help people identify, deal with, and prevent mental illness" [8]. A few years later, Jorm [9] noted that the knowledge about mental illness in MHL is not simply about the knowledge obtained from the classroom but should be more about the knowledge of behavioral tendencies that can promote the mental health of oneself or others, and include the skills of helping others to seek professional psychological help. Therefore, the construct of MHL has been revised to the knowledge of mental illness prevention, timely identification of developing mental illness, knowledge on seeking appropriate help and treatment, knowledge on effective self-help for milder mental illness, and first-aid skills that can help those who are prone to or have mental illness. However, it is still rather limited to coping with mental illness. Later on, researchers [10] sorted out and simplified its dimensions, forming a conceptual structure of three dimensions: recognition, knowledge, and attitude toward mental illness. As the research deepened, some scholars began to incorporate the content of mental health promotion into MHL [11], which received support from some scholars [12].

MHL and mental health promotion

With the understanding that MHL encompasses decreasing mental health stigma and increasing help-seeking effectiveness, MHL has been considered as the first step toward mental health promotion. According to the World Health Organization (WHO), as stated in its “Comprehensive Mental Health Action Plan 2013-2030”, MHL is one of the strategies for mental health promotion and prevention [13]. Evidence has shown that the MHL of the general public has been gradually increasing but is still considered low or moderate. Hence, efforts must be taken to enhance MHL across demographics, particularly in vulnerable populations like the youth [14,15]. For mental health promotion to be effective, protective factors must be developed and supportive environments like educational institutions must be present. Mental health promotion must go beyond the health sector and penetrate different sectors such as education [16-19]. Recently, children and youth are some of the main priorities in mental health promotion [15].

MHL is seen as an important factor affecting individual mental health, and its impact on mental health is mainly achieved through the following three pathways: first, a high level of MHL can enhance the ability to identify, diagnose, and prevent mental illness; second, a low level of MHL will reduce the timeliness of individuals seeking psychological help and the effectiveness of mental health services; third, a lack of MHL will increase the risk of developing mental illness or other long-term adverse outcomes [17].

International research on MHL

Qi et al. [18] found that international MHL research is generally on the rise, and Australia, the United States, and the United Kingdom are the countries at the forefront of MHL research, mostly covering topics on MHL promotion. Australian studies mostly focus on activities that would help improve one's MHL. Studies in the US and the UK seem to focus more on measuring people's levels of MHL. The US tends to have a better understanding of mental illness, while the UK is relatively weaker [19-21]. This may be due to differences in economic development levels. 

In terms of recommendations for improving MHL, a higher proportion of British participants believed that professional help would be useful as compared to some Asian counterparts in Hong Kong and Malaysia [22]. In developing countries like Indonesia, the MHL of healthcare professionals is emphasized [23].

MHL research in China

A scoping review found that research on MHL has grown increasingly in China in terms of frequency and geographic coverage from 1997 to 2018 [24]. From among the 350 peer-reviewed studies in this review, the majority (313, 89.4%) of these studies were from Chinese language journals while the rest were published in English language journals. Most (303, 86.6%) yielded survey results and the rest examined the effectiveness of MHL interventions. The assessment of mental-health-related knowledge and beliefs was the focus of most MHL research in China, and the development and evaluation of MHL interventions were given less attention by scholars in the past. General awareness of mental health and suicide as related to MHL were the topics most commonly studied, and specific illnesses apart from some that did cover depression, anxiety, and psychosis received less focus. Most (97, 80.2%) of the measurement tools on MHL were developed in China and 57.8% were not able to provide sufficient information on their psychometric properties. Hence, this review emphasized the need to implement more interventions to enhance MHL in China, utilize more sophisticated research methods, and validate MHL measurement tools. In response to this, there is actually a recent effort to develop a standardized measure of MHL in China. For example, in the study by Wu et al., a measurement tool for MHL suitable for China was developed, the Mental Health Literacy Questionnaire (MHLQ). The study summarized the six-factor structure of mental health knowledge, mental illness knowledge, attitudes toward mental health, attitudes toward mental illness, behaviors that promote mental health, and behaviors that cope with mental illness. The six-factor MHLQ largely supports the expanded theoretical concept of MHL in the Chinese context. The study pointed out that further verification of the factor structure and examination of measurement invariance are needed in the future [25]. 

Another study worth mentioning was in 2019, a nationwide comprehensive assessment of the Chinese public’s ability to recognize mental disorders and recommend help resources, which was conducted in order to address the nation’s treatment gap [26]. Data was collected as part of the Stigma in Global Context-Mental Health Study (SGC-MHS), aiming to understand the stigma level among 16 countries in the Global North and Global South regions. A cross-sectional multistage probability sampling was also used to choose adult samples who were nationally representative of Mainland China. It was reported that apart from depression which is relatively higher and most accurate in urbanized areas, there is generally a low recognition of mental health concerns. When a mental illness is perceived to be severe, this increases the likelihood of being recommended for care and treatment by different health providers. Being able to recognize a mental health concern also specifically increases the likelihood of being endorsed to a psychiatrist and other mental health professionals. Mental illnesses with neurobiological characteristics were also mostly endorsed to general or specialized physicians as opposed to mental health professionals. The study highlighted the importance of continuous initiatives on mental health promotion, especially in rural areas, and specific mental illnesses like schizophrenia. Increased mental illness recognition and addressing challenges of people knowledgeable of the neurobiological sources of mental illnesses may serve as an important strategy to decrease the significant mental health treatment gap in China.

Cultural factors

One major concern with mental health education in China is that cultural factors remain a contributor to people’s misconceptions or misinformation about mental health and illnesses [27]. 

In traditional Chinese culture, people’s understanding of health refers only to physical health. With the development of society, Chinese people have gradually realized that overall health includes mental health. For a long time, people held a stigma against mental illness, that is, people have negative evaluations and negative emotional experiences toward people with mental illness, and there is discrimination, especially among teenagers [28]. With the development of modern society, the fast pace, and the impact of foreign cultures, many young people pay more attention to material enjoyment and personal achievements. To help young people better adapt to society and pay attention to their physical and mental health, it is necessary to carry out MHL education, and equip them with mental health knowledge, skills, and attitudes, to improve their physical and mental health [29].

MHL of college students

Particularly in the demographic of college students, their levels of MHL have been largely explored as related to different variables. In recent studies, for example, 1213 college students from an urban public university in the US were asked to participate in an assessment of MHL alongside demographic, psychological, and academic factors. Results revealed that variation in MHL was primarily attributed to taking a clinical psychology course, followed by having psychology as their major. Also, regardless of whether they scored higher or lower in MHL, having increased knowledge and awareness of mental health was key to overall psychological well-being [30].

Another study in the US in 2022 explored relationships between MHL, help-seeking, and mental health outcomes [19]. Results revealed that female college students had significantly higher MHL scores than male students, and students with previous mental illness diagnoses scored higher in MHL too than those with no prior diagnosis. MHL and self-compassion were also significantly positively correlated. In addition, the same results were found in 2024 when MHL was examined alongside psychological distress and help-seeking attitudes among 357 local and international college students in Australia. Results revealed that while student group type does not predict help-seeking intentions for emotional concerns or psychological distress, international students were reported to have lower help-seeking attitudes, help-seeking intentions for suicidal ideation, and MHL scores [31]. Both studies underscored the need to design interventions that are culturally competent and tailored to diverse student populations.

MHL of Chinese college students

Research into the mental health of Chinese college students emerged quite late [32] and has a rather scarce base. More recently, this research area has focused primarily on defining and gauging the comprehension of mental health [33]. Researchers have noted that there is insufficient exploration into the current status of college students' understanding of MHL [5,32]. Most existing studies also use purely quantitative research methods [4]. Hence, the use of other research approaches like qualitative or mixed-methods research holds significant potential for the thorough investigation and exploration of Chinese college students’ MHL.

Just like Western studies, recent MHL research for Chinese college students has been studied about other personal, psychological, and academic factors. Chinese students with low levels of MHL were found to be more likely to have mental health problems than students with high levels of MHL. Similarly, college students with low MHL levels are more likely to cause certain psychological problems when they encounter difficulties that may lead to extreme incidents such as suicide [34]. A recent study found that the MHL of college students is generally low, but the students who served as peer counselors, class counselors, or dormitory psychological information officers in schools have significantly higher MHL, specifically, positive MHL, as compared to other students. This gives us an important revelation that increasing the MHL training of this group of students may better promote the MHL of all students [34]. Therefore, college students should not only have basic knowledge and skills in mental health but also have the concept of helping others acquire the same. Colleges and universities should take the improvement of the MHL of all college students as the fundamental starting point of mental health education, strengthen the popularization of mental health knowledge, establish a new and effective mechanism for psychological education, and further promote mental health education in colleges and universities [32,34].

College students are the pillars of future society and shoulder the important task of leading modernization efforts. Student-centered learning strategies are of great significance to students [35]. Mental health is not limited to affecting the growth and maturity of individuals, but also has a profound impact on national progress and future development [5]. Therefore, educational institutions must pay attention to policy guidance. Since the mid-1980s, relevant policies have been introduced one after another, and mental health education has received more and more attention [36]. For example, research on the factors affecting college students' mental health education has become more and more extensive [37]. The “Healthy China Action Promotion Committee” proposed that by 2030, the mental health level of Chinese citizens must have increased to 30%. To do so, efforts should be made to improve their mental health level, slow down the rising trend of mental-related diseases, and actively respond to the current prominent mental health problems [38]. On February 21, 2024, the first plenary meeting of the National Student Mental Health Steering Committee was held in Beijing to conduct an in-depth analysis of the situation and tasks of promoting the improvement of students' mental health.

Mental health education

Educational institutions hold a key position in implementing interventions to address mental health concerns and improve the MHL of college students. While a very strict definition of mental health education currently seems to not exist, it is loosely described as activities and initiatives that aim to improve one’s MHL. Mental health education needs to strengthen its practicality, ensure that it can meet the diverse psychological needs of college students, and adapt to real-life situations. This means that mental health education should not only focus on the teaching and learning of theoretical knowledge, but also on commitment to transforming theory into practical skills, and cultivate individuals' ability to cope with life pressures, self-adjustment, autonomy, and help others solve psychological problems [39]. The improvement of college students' MHL effectively fills the gap in the implementation of college students' mental health education process in previous studies. In the present times, facing the new situation and new characteristics of college students' mental health, cultivating college students' MHL, and improving the effectiveness of mental health education have become important contents of China's mental health education research.

The study found that educational campaigns may have some positive effects on depression stigma [40].

Mental health education in China

After years of development, the mental health education of colleges and universities in China has achieved significant results and shows the characteristics of distinctiveness, standardization, and quality [37].

Chinese college students’ mental health education implements a "four-in-one" work pattern of education and teaching, practical activities, consulting services, and preventive intervention.

Barriers to effective implementation

There are many problems in the development of mental health education, the most important of which is trying to align it with China's actual mental health needs and situation [36].

In terms of its teachers, there are also several documented challenges like teachers’ increasing professional pressure that leads to their own mental health problems which seriously affects the students [41]. 

Another challenge is the lack of variety in teaching methods, with courses mainly focused on theory. In recent years, the development of educational technology has triggered a revolution in classroom teaching, changed the time and space constraints of classroom teaching, and changed the roles of teachers and students. Teachers have changed from being mere knowledge transmitters to being the guides of students’ learning; students have changed from being passive to active learners. MOOC, flipped classroom, micro-class, micro-video, and other technologies have been cleverly introduced into classroom teaching, but they only stay in form. Teachers' educational concepts have not changed. The traditional teacher-centered teaching model, that is, the teaching-oriented teaching method, still dominates [42]. As humans enter the information age, the disadvantages of the teaching-centered classroom teaching model are becoming increasingly prominent, such as it being difficult to mobilize students' enthusiasm, and students being unwilling or unwilling to learn, resulting in distraction in class. There are problems such as too much content, too few class hours, large class teaching, and limited teaching space in the mental health education courses of ordinary universities [27].

An attempt at effective implementation

Combine theory with practice, optimize the teaching level of teachers, course content, and teaching objectives, actively carry out research, and improve students' innovation ability [43].

With the continuous development of higher education, explore the pertinence and effectiveness of mental health education in the new media environment [44]. Due to the influence of cultural concepts, breakthroughs in China's mental health services should also be given priority in teaching [45]. At present, Professor Zhang [46] proposed to be student-oriented and infiltrate psychological education into daily work.

MHL plays a crucial role in promoting the development of mental health education in China. Specific manifestations of MHL can improve the high-quality development of mental health education and inspire the endogenous development of school teachers' and students' mental health. A good mental health education environment must be provided [47]. With MHL as the basic goal of college students' mental health education, the problems encountered by mental health education will be effectively addressed [33].

Implications of MHL to mental health education in China

China's "Guidelines on Strengthening Mental Health Services" (National Health and Disease Control) regards improving the MHL of the people as an important part of the current construction of a physical and cultural civilization society and puts forward the basic goal of mental health services that by 2030, the MHL of the whole population will have generally improved [38]. China is in a period of rapid economic and social transformation, people's pace of life has significantly accelerated, and mental health problems are becoming increasingly prominent. At present, the mental health issues of the Chinese public are characterized by high prevalence and low rates of medical treatment, and improving the public's MHL is an important way to solve the above problems. Improving the MHL of college students, a special group, is not only conducive to strengthening their health level and reducing the incidence of students with psychological problems but also an important action at the national level to improve the mental health of all.

With the evolution of the times and the profound changes in social structure, the construct of MHL has been unprecedentedly enriched and deepened. Most researchers conducted surveys on this, and research and analysis on college students' MHL are based on questionnaires translated from foreign countries [5,48]. Ming and Chen [33] pointed out that through qualitative research, the public and experts' understanding of MHL was gathered, and combined with a literature review, the content structure and indicator system of MHL was initially constructed. The system was then verified and optimized using expert judgment, and a highly reliable and valid measurement tool was developed. The MHL of college students in the present context is a multidimensional and multilevel comprehensive construct that covers many aspects such as knowledge and concepts, healthy lifestyle, basic skills, and emotional attitudes.

The key way to improve college students' MHL is mainly through educational intervention [31]. Many researchers have shown that mental health education can improve college students' MHL [49-52]. Zeng et al. [34] found that students with low MHL are more likely to have various mental health problems than those with high literacy, and may even experience extreme events such as suicide or harming others when facing life setbacks. Therefore, actively carrying out targeted mental health education activities is crucial to improving college students' MHL and can effectively improve the psychological state of students with psychological distress.

Wu et al. [53] found that the three factors of psychological resilience, emotional control, self-plasticity, and coping flexibility, were all significant influencing factors of positive coping styles. Jing et al. [54] found that the MHL of college students in Beijing was generally higher than that of their peers, and was also affected by majors, place of origin, and health-related course selection. Lai et al. [32] showed that improving MHL can promote mental health at the individual and public levels. Jiang et al. [5] further confirmed that the current status of MHL among Chinese college students is not optimistic, which is inseparable from the significant influence of factors such as social environment, family education, and personal growth experience.

Research results show that college students' mental health education and training have a significant impact on their mental health level, and this impact has a far-reaching impact [55]. Liu et al. [56] emphasized that mental health level is a breakthrough in solving mental health problems.

Recommendations and future directions

To comprehensively improve the MHL of college students, mental health education in Chinese colleges and universities should shift from a rehabilitative to a preventative approach, ultimately guiding students toward overall self-improvement. From merely addressing immediate psychological problems, there is a need to systematically develop a mental health education framework that includes dissemination of knowledge, promotion of healthy behaviors, relevant skills training, and shaping of positive attitudes all in the present context. Furthermore, developing the MHL of college students through mental health education should emphasize "literacy” -- understanding mental health concepts, possessing relevant knowledge, developing skills, and shaping positive attitudes.

## Conclusions

In summary, developing MHL for Chinese college students in modern times is a critical task for educators and educational institutions, as the promotion of mental health has implications that extend beyond improving students’ mental health and also for others and society. 

This review emphasizes the need for a comprehensive approach to MHL, which includes sharing mental health information, encouraging healthy behaviors, developing coping mechanisms, and reducing stigma. Incorporating evidence-based mental health education into the academic curriculum is crucial for creating a proactive and supportive atmosphere in colleges and universities.

To fill the gaps in MHL research, especially in China, more advanced methodologies are necessary, such as more qualitative and mixed-methods studies, to gain a clearer understanding of students' needs. Additionally, prioritizing culturally adapted interventions that address the specific challenges related to traditional beliefs, stigma, and the differences in resources between urban and rural regions is critical.

Looking forward, a systematic shift from reactive to preventive mental health education is needed. By increasing the MHL of the students through mental health education, China can foster a generation of individuals with resilience and contribution to societal well-being. Researchers, educators, and policymakers must work collaboratively to ensure MHL efforts are comprehensive, accessible, and effective, paving the way for a healthier, more informed society.
